# Scorpion Venom: Detriments and Benefits

**DOI:** 10.3390/biomedicines8050118

**Published:** 2020-05-12

**Authors:** Shirin Ahmadi, Julius M. Knerr, Lídia Argemi, Karla C. F. Bordon, Manuela B. Pucca, Felipe A. Cerni, Eliane C. Arantes, Figen Çalışkan, Andreas H. Laustsen

**Affiliations:** 1 Department of Biotechnology and Biomedicine, Technical University of Denmark, DK-2800 Kongens Lyngby, Denmark; juliusknerr@aol.com (J.M.K.); lidia.argemimuntadas@gmail.com (L.A.); manu.pucca@ufrr.br (M.B.P.); felipe_cerni@hotmail.com (F.A.C.); 2 Department of Biotechnology and Biosafety, Graduate School of Natural and Applied Sciences, Eşkisehir Osmangazi University, TR-26040 Eşkisehir, Turkey; fcalis@ogu.edu.tr; 3 Department of BioMolecular Sciences, School of Pharmaceutical Sciences of Ribeirão Preto, University of São Paulo, Ribeirão Preto—São Paulo 14040-903, Brazil; karla@fcfrp.usp.br (K.C.F.B.); ecabraga@fcfrp.usp.br (E.C.A.); 4 Medical School, Federal University of Roraima, Boa Vista, Roraima 69310-000, Brazil; 5 Department of Biology, Faculty of Science and Letters, Eskisehir Osmangazi University, TR-26040 Eskisehir, Turkey

**Keywords:** scorpion venom, potassium channel toxins, calcins, scorpionism, fungicide, parasiticide, bradykinin potentiating peptide, analgesics, antivenom

## Abstract

Scorpion venom may cause severe medical complications and untimely death if injected into the human body. Neurotoxins are the main components of scorpion venom that are known to be responsible for the pathological manifestations of envenoming. Besides neurotoxins, a wide range of other bioactive molecules can be found in scorpion venoms. Advances in separation, characterization, and biotechnological approaches have enabled not only the development of more effective treatments against scorpion envenomings, but have also led to the discovery of several scorpion venom peptides with interesting therapeutic properties. Thus, scorpion venom may not only be a medical threat to human health, but could prove to be a valuable source of bioactive molecules that may serve as leads for the development of new therapies against current and emerging diseases. This review presents both the detrimental and beneficial properties of scorpion venom toxins and discusses the newest advances within the development of novel therapies against scorpion envenoming and the therapeutic perspectives for scorpion toxins in drug discovery.

## 1. Introduction

According to national public health data, about 1.5 million scorpion envenomings, resulting in 2000–3000 deaths, are recorded annually worldwide [1,2]. While large regions in the Northern Hemisphere, such as the United States, Canada, Europe, and Russia, as well as Australia in the Southern Hemisphere are not associated with severe scorpionism [3], more than two billion people living in northern Saharan Africa, African Sahel, South Africa, Near and Middle East, southern India, eastern Andes, Mexico, and South America are at risk of being stung by scorpions [1]. Climate change together with urban expansion and poor city sanitation management in many of these areas have increased the likelihood of encountering scorpions. For instance, in Brazil alone, malign incidences with scorpions have nearly doubled from 64,000 to 124,000 annual envenoming cases since 2012 [4].

To date, over 2000 scorpion species have been described. The vast majority of the scorpion species that are dangerous to humans belong to the Buthidae family [5], but some species in the families of Scorpionidae and Hemiscorpiidae have also been classified as harmful [6,7]. Geographical distributions of most of these medically significant species correlate with the local prevalence of scorpionism. Hence, the density of dangerous species is especially high in northern Africa, Iran, Saudi Arabia, Brazil, Mexico, and Venezuela [3,8,9]. Local pain is often the first symptom of scorpion envenoming, which may set in only minutes after a sting has occurred. Depending on the scorpion species, the symptoms can progress to severe complications over the course of a few hours. Inducing a massive release of neurotransmitters, scorpion venom neurotoxins usually cause sweating, nausea, vomiting, hypersalivation, restlessness, and, in more severe cases, arrhythmia, unconsciousness, and heart failure, which may lead to death [10]. However, in spite of the hazardous and life-threatening effects of scorpion envenoming, therapeutic properties of scorpion body parts and venoms in ancient medicine have been utilized by humans for thousands of years [10]. Nowadays, the potential therapeutic value of different scorpion venom compounds is being increasingly investigated, as these compounds may represent promising leads for the development of new pharmaceuticals.

In this review, we survey the field of scorpion venom research from different angles focusing both on the detrimental and beneficial properties of scorpion venom toxins. First, scorpion venom compounds together with clinical manifestations and symptoms of different levels of scorpionism are introduced. Then, currently available treatments and research into new alternatives, i.e., next-generation antivenoms, are discussed. Finally, the latest reported results from the scientific literature focusing on the widespread potential applications of scorpion venom compounds are presented.

## 2. Scorpion Venom Compounds

Scorpions use their venom to defend against predators and to capture prey. The composition of scorpion venom is highly complex and heterogeneous. Up until now, small scorpion venom peptides are the most studied compounds, mainly due to their diversity and broad pharmacological properties. Accordingly to their structure, these small peptides are classified into three large superfamilies: peptides containing cysteine-stabilized (CS) α/β motifs, calcins, and non-disulfide bridged peptides (NDBPs) [11]. However, enzymes (larger proteins), mixtures of inorganic salts, free amino acids, nucleotides, amines, and lipids are also found in scorpion venom [12].

### 2.1. Peptides Containing CS α/β Motifs

**Figure 1:**
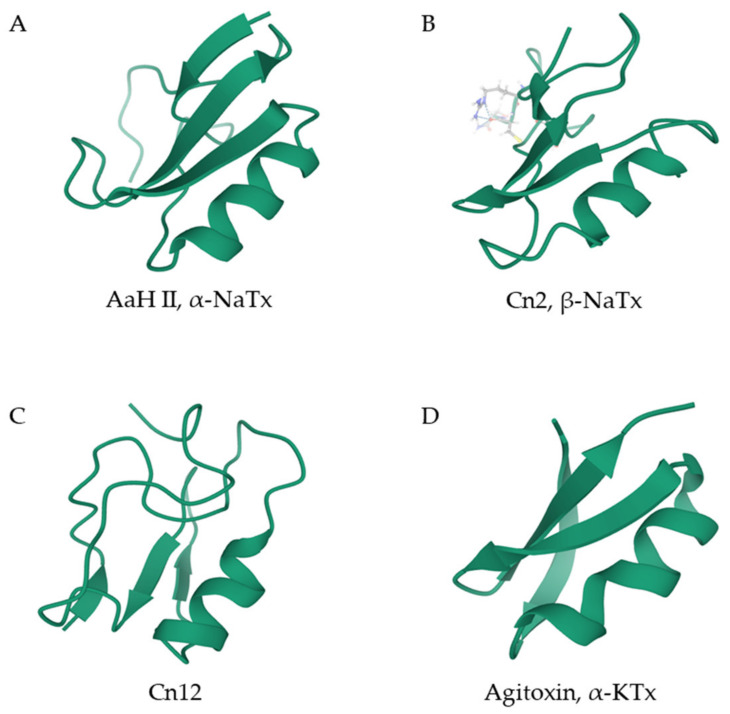
Ribbon diagrams of the 3D structure of selected scorpion venom peptides containing the cysteine-stabilized (CS) α/β motif. (**A**) AaHII from *Androctonus australis* is a classical α-NaTx. (**B**) Cn2 from *Centruroides noxius* venom is a classical β-NaTx. (**C**) Cn12, also from *C. noxius* venom, shows structural resemblance to β-NaTxs, but exhibits an α-NaTx function. (**D**) Agitoxin 1 from *Leiurus hebraeus* (previously *L. quinquestriatus hebraeus*) is an α-KTx toxin. The Protein Database accession numbers are 1PTX for AhHII; 1CN2 for Cn2, 1PE4 for Cn12, and 1AGT for agitoxin 1. KTx: potassium channel toxins, NaTx: sodium channel toxins.

These peptides consist of an α-helix joined to a double or triple-stranded β-sheet via a disulfide bridge (Figure 1) [12]. These molecules present two completely conserved disulfide bonds in the C_i_–C_j_ and C_i+4_–C_j+2_ positions; although some of them also exhibit an extra link connecting the two endings of the peptide chain [11]. All scorpion peptides containing CS α/β motifs act in a similar way. Their interaction with ion channels result in blocking or modulation of the normal mode of action of these channels [12]. Members of this superfamily can be subdivided into long or short scorpion toxin families, corresponding to their respective structures.

#### 2.1.1. Long Scorpion Toxins

Peptides from the long scorpion toxin superfamily are 55–76 residue-long molecules with generally four disulfide bridges [13]. Due to their mode of action, they can also be called sodium channel toxins (NaTxs), as their main targets are sodium ion channels [12]. This family can be further divided into two groups, α and β-NaTxs, depending on their specific interaction with the voltage-gated Na^+^ channels (Figure 1A,B). α-NaTxs block site 3 of sodium ion channels, and therefore inhibit the inactivation of the channels and prolong their action potential. In contrast, β-NaTxs interact with site 4 of Na^+^ channels and shift the activation voltage of the channels to a more negative potential, which results in channel inactivation (opposite to the effects of site 3 toxins) [14]. It is noteworthy that not all NaTxs can be assigned to these two groups. For example, Cn12, from *Centruroides noxius*, and Ts2, from *Tityus serrulatus* venom, structurally resemble a β-NaTx but exhibit an α-NaTx effect (Figure 1C) [15,16]. In addition, AaH IT4, a toxin from *Androctonus australis hector*, displays both α and β-NaTx effects [11].

#### 2.1.2. Short Scorpion Toxins

The short scorpion toxin family is composed of peptides of 23–64 residues in length with three or four disulfide bridges. These peptides, also known as potassium channel toxins (KTxs), mainly act as potassium ion channel blockers (Figure 1D) [17]. Considering their sequences and cysteine pairs, KTxs can be divided into α, β, γ, κ, δ, λ, and ε-KTx groups [18,19]. The α-KTx group, which is considered to be the largest subgroup of the short scorpion toxin family, contains 23–42 residue-long peptides with three or four disulfide bridges [20]. The β-KTx group comprises longer chain peptides of 50–57 residues in length [21]. The γ-KTx group can be found in the genera *Centruroides*, *Mesobuthus,* and *Buthus*, and it mainly blocks the human *Ether-à-go-go*-Related Gene (hERG) channels [22]. Instead of having a CS α/β structure, similar to the α, β, and γ-KTx subfamilies, toxins of the κ-KTx group are composed of two parallel short α-helices connected by a β-turn that is stabilized by two disulfide bridges, yet their interaction with potassium ion channels is similar to that of the α-KTx group [23].

Although the δ, λ, and ε-KTx groups do not contain a CS α/β motif, they are mentioned in the continuation of other KTxs. The δ-KTx group contains a Kunitz-type structural fold with a double-stranded antiparallel β-sheet flanked by an α-helix in both the C-terminal and N-terminal segments. Since the Kunitz-type structural folds are the active domains of proteins that inhibit the function of serine proteases, δ-KTxs exert both protease and potassium channel-inhibiting properties [24]. The λ-KTx group, similar to calcins, contain an inhibitor cystine knot (ICK) motif (see Section 2.2) that contains a triple-stranded antiparallel β-sheet stabilized by three cystine linkages [25,26]. The ε-KTx group has recently been defined and, so far, it has just two members, Ts11 and Ts12 from *T. serrulatus* venom. Ts11 shows less than 50% identity with KTxs from other subfamilies. Ts11, similar to λ -KTxs, contains an ICK motif. However, λ-KTxs possess only three disulfide bridges, while Ts11 has four disulfide bridges assembled in a unique pattern [19].

### 2.2. Calcins

This small, but growing, family of scorpion toxins consists of calcium channel-modulating peptides, such as imperacalcin (imperatoxin), maurocalcin, hemicalcin, hadrucalcin, opicalcin, urocalcin, and vejocalcin [27]. Sharing high sequence similarity (>78% identity), calcins include an ICK motif stabilized by three disulfide bridges [28]. Calcins mainly act as agonists of ryanodine receptors (RyRs), which are intracellular ligand-activated calcium channels that are found in endoplasmic/sarcoplasmic reticulum membranes. RyRs play an essential role during excitation–contraction coupling in cardiac and skeletal muscles by releasing Ca^2+^ from intracellular reservoirs [29]. In general, calcins induce long-lasting subconductance states on the RyR channels, which lead to an increase in the intracellular Ca^2+^ level and subsequently contractile paralysis [30].

Calcins also present the ability to pass through cell membranes without causing their lysis [31]. It has been hypothesized that the clustering of positively charged, basic residues on one side of the calcins gives them a dipole moment that possibly interacts with negatively charged membrane lipid rafts, such as gangliosides. Once these toxins interact with the outer membrane, interaction between the hydrophobic regions of the toxin and the inner membrane is favored, and the toxin is transiently translocated. Further electrostatic interactions with negatively charged molecules from the cytoplasm trigger the entrance of calcins into the cell without disrupting its membrane [32]. This feature makes the calcins excellent candidates for intracellular drug delivery, since they can enter cells without disrupting them, even when large membrane-impermeable molecules are conjugated to them [33].

A calcium channel modulator, distinct from the toxins that act on RyRs was recently identified through transcriptome analysis of *T. serrulatus* and designated as a cell-penetrating peptide (CPP)-Ts. The synthetic CPP-Ts is the first described scorpion toxin that activates Ca^2+^ signaling through the nuclear inositol 1,4,5-trisphosphate receptors. This toxin, together with the calcium channel toxin-like BmCa1 from *Mesobuthus martensii*, forms a new subfamily of calcium toxins and shows promising anticancer effects (see Section 4.8) [34].

It is noteworthy that calcins are not the only cell-penetrating scorpion toxins. The transient receptor potential cation channel subfamily V, member 1 (TRPV1) is a chemosensory ion channel, which is also known as the wasabi receptor. Generally, TRPV1 is activated through a unique mechanism involving the covalent modification of specific cysteine residues located within the channel’s cytoplasmic N-terminus. Recently, it has been reported that Wasabi Receptor Toxin (WaTx), from *Urodacus manicatus* venom, is capable of activating this receptor. This means that WaTx can cross the plasma membrane and bind to the same allosteric nexus that is covalently modified by other agonists [35].

### 2.3. Non-Disulfide Bridged Peptides (NDBPs)

NDBPs are small, 13–56 amino acid-long peptides with a very heterogeneous composition. Compared to scorpion peptides with disulfide bridges, NDBPs do not present a conserved or predictable structure-function relationship [36]. Most of these peptides are cationic molecules that display notable structural flexibility. In aqueous solutions, these peptides exhibit a random coil conformation. However, under membrane-mimicking environments, such as 50%–60% of aqueous trifluoroethanol, they readily adopt an amphipathic α-helical structure [37]. This characteristic enables them to interact with a broad spectrum of biological targets; however, they do not have any known specific molecular targets [38,39].

### 2.4. Enzymes

Few enzymes have been found in scorpion venoms, in part because up until recently, interest has been focused on small proteins and peptides. However, during the past years, hyaluronidases, phospholipases, and metalloproteases, among other enzymes, have been detected in venoms of different scorpion species.

Different hyaluronidases have been identified in different families of scorpions, including Buthidae, Bothriuridae, and Urodacidae [40]. It is known that these enzymes potentiate the toxicity of venom by disrupting the integrity of the extracellular matrix and connective tissues surrounding blood vessels at sting point, and they thereby ease the systemic diffusion of other relevant scorpion toxins [41]. It has recently been demonstrated that hyaluronidases also play an essential role in venom distribution from the bloodstream to the target organs [42]. The same study also indicated that the neutralization of hyaluronidases could be considered as a first-aid strategy in scorpion envenoming treatment.

Phospholipases are known to be potent hemolytic agents, as they disrupt cell membranes by hydrolyzing phospholipids. They can also cause tissue necrosis and hemorrhages. Phospholipase activity has been detected in several scorpion species, including *Opisthacanthus cayaporum* [43] and *Heterometrus laoticus* [44].

Generally, venoms from *Tityus* species exhibit significant proteolytic activity, and the first scorpion metalloprotease was discovered in the venom of *T. serrulatus* [45]. Metalloproteases and serine proteases have also been detected in *T. discrepans* venom [46] and, apart from *Tityus* species, in the venom of *Hemiscorpius lepturus* [47]. It is believed that proteases play a key role in activating toxin precursors through post-translational modifications [48]. In addition, these enzymes inhibit platelet aggregation, modulate cytokine production, and activate the complement system [49,50]. Altogether, these effects facilitate the diffusion of scorpion venom toxins via the degradation of matrix proteins.

### 2.5. Other Venom Compounds

Only a very limited number of studies on non-peptidic scorpion venom compounds have been reported. However, it has been shown that the venoms of some scorpions, including *M. tamulus*, contain serotonin, which is a monoamine that may cause vomiting and considerable local pain in scorpion envenomings [51]. The metal and salt composition of some scorpion species have also been evaluated by Al-Asmari et al. [52]. In their study, they found copper, zinc, calcium, magnesium, iron, lead, manganese, arsenic, and nickel ions in the venom of *A. bicolor*, *A. crassicauda*, and *Leiurus quinquestriatus*. They suggested that these components are associated with enzyme activity, as they probably act as enzyme cofactors. In addition, two 1,4-benzoquinone compounds, with antimicrobial activity, have recently been isolated from the venom of *Diplocentrus melici*, a scorpion species endemic to Mexico [53] (see Table 1). Moreover, it has been reported that one of the low-molecular-weight compounds with anticoagulant activity isolated from the *Heterometrus laoticus* venom is adenosine, which is a well-known inhibitor of platelet aggregation [54,55].

## 3. Detriments of Scorpion Venom: Scorpion Envenoming

**Figure 2:**
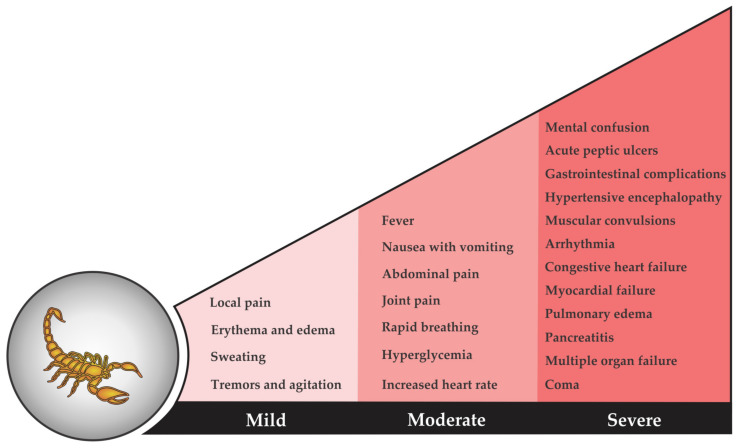
Clinical manifestations and symptoms of mild, moderate, and severe scorpion envenomings. Typical symptoms of mild stings last for minutes to hours and include great local pain, a reddened and swollen site of sting (erythema and edema), numbness, sweating, body tremors, and agitation. More intense stings from scorpions with venom containing cytolytic toxins may result in blood blisters, hemorrhages, and necrosis of the surrounding tissue. In moderate envenoming cases, the body additionally reacts with fever, abdominal and joint pain, hyperglycemia, abnormally rapid breathing, increased heart rate, and nausea with vomiting. These symptoms can reside for days and are mostly caused by neurotoxins: Na^+^, K^+^, and Ca^2+^ ion channel modulators. Neurotoxins can also cause severe envenomings, which can lead to cardiovascular, neurological, pulmonary, and/or gastrointestinal complications, such as pulmonary edema, myocardial failure, arrhythmia, congestive heart failure, extreme muscular convulsion, hypertensive encephalopathy, acute peptic ulcers, pancreatitis and lethal multiple organ failure, mental confusion, and coma. After several days, most victims of lethal scorpion stings die from cardiac or respiratory failure.

Scorpion envenomings can cause severe pathological effects and even death in humans. The intensity of an envenoming usually depends on the victim’s sensitivity and body mass, the anatomical location of the sting, the amount of injected venom, and the scorpion species. Commonly, based on the severity of symptoms, scorpion envenomings are classified into three levels: mild, moderate, and severe (Figure 2) [56,57]. Mild envenomings result in local inflammatory reactions, whereas moderate and severe envenomings may provoke lethal systemic responses.

### 3.1. Scorpion Envenoming Treatment

**Figure 3:**
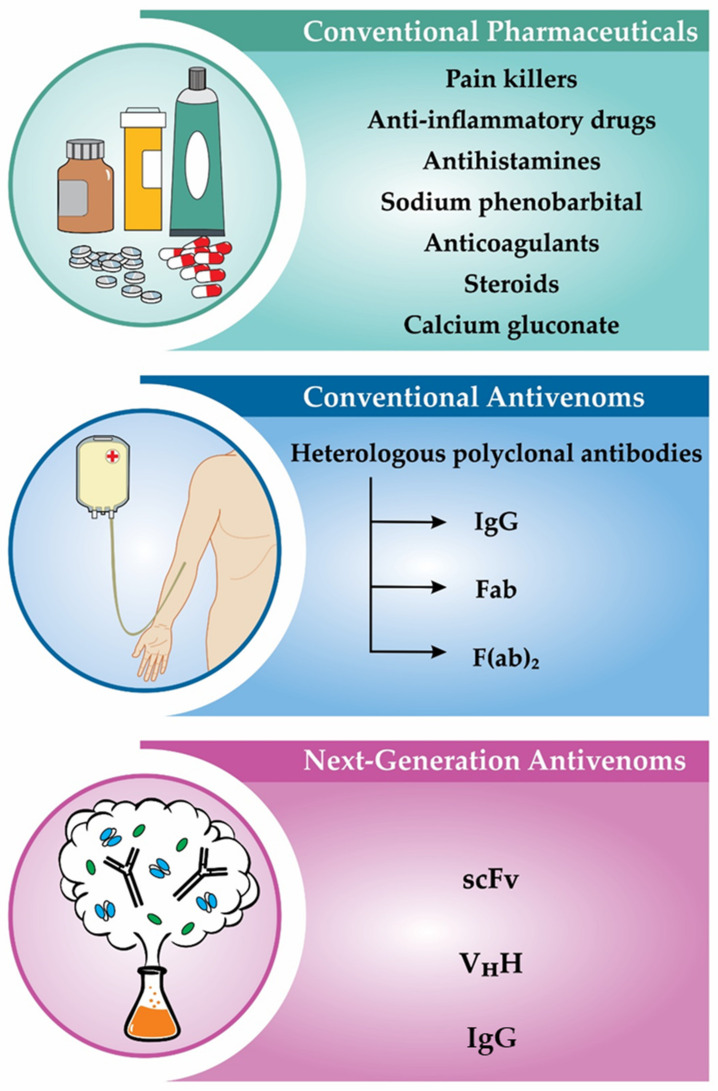
Scorpion envenoming treatments. Conventional pharmaceuticals are used for mild envenomings, while antivenom therapy is applied in moderate and severe cases. Recombinant antivenoms are suggested to have higher therapeutic value over the conventional antivenoms and may become the future mainstay of treatment.

Generally, the first treatment strategies that are undertaken after an envenoming event focus on pain relief and possibly intravenous hydration to decrease the negative effects of strong salivation and sweating. In order to relieve acute pain after a scorpion sting, either cooling by ice or intravenous injection of paracetamol or nonsteroidal anti-inflammatory drugs, such as diclofenac and indomethacin, or topical administration of lidocaine cream at the site of the sting can be used [58]. However, it is not surprising that the analgesic effect of lidocaine might be superior to the former treatments [59]. Further substances that are considered for application in envenoming cases are prazosin (which counteracts catecholamine-induced hypertension) [60], antihistamines and steroids (which reduce inflammatory responses) [61], sodium phenobarbital (which prevents convulsions and lung edema) [8,62], and calcium gluconate (which eases muscle spasms) [63]. While partially being used in practice, there does not seem to be a general consensus on the efficacy and possible adverse effects of these treatment options. Yet, in the case of prazosin, it has been shown that the mortality and the mean residence time in the hospital could be significantly reduced by the administration of two doses of the drug, one immediate and one three hours after the envenoming incident [64]. Additionally, small molecules, such as heparin, ethylenediaminetetraacetic acid EDTA, and aristolochic acid have been shown to neutralize scorpion venom enzymes, such as hyaluronidases, phospholipases A_2_, and metalloproteases. Thus, these molecules might be considered as a starting point for the development of future treatments [5]. In more severe envenoming cases, antivenoms are employed to neutralize the venom and diminish the morbidity and mortality of scorpion stings (Figure 3).

#### 3.1.1. Conventional Plasma-Derived Antivenoms

Conventional plasma-derived antivenoms are produced by the purification (and digestion) of polyclonal immunoglobulin G (IgG) molecules harvested from the plasma of hyperimmunized animals, such as horses or sheep. These polyclonal antivenoms can cause severe adverse reactions due to their heterologous origin [65] and are known from the field of snakebite envenoming to generally possess low percentages of venom-neutralizing antibodies [66,67]. Moreover, conventional antivenoms may have limited efficacy against the medically most important toxins, such as small neurotoxins, as these toxins are often weakly immunogenic and therefore fail to raise a strong antibody response in the production animal [68]. Nonetheless, the administration of these types of antivenoms is effective for clinical use and has been life-saving since the 1900s. Thus, plasma-derived antivenoms are still the standard of treatment for systemic scorpion envenomings [69].

Recently, in pursuit of finding alternatives to equine IgGs for scorpion envenoming treatment, strategies involving avian egg-yolk-derived immunoglobulin Ys (IgYs) have been developed [70]. In contrast to equine IgGs, avian IgY antibodies are obtained noninvasively from egg yolks from laying hens that have been immunized with scorpion venom. These IgY molecules have been argued to activate the mammalian complement system much less than animal-derived IgGs and not interact with rheumatoid factors. They are produced in amounts comparable to those found in the plasma of large mammals and have been argued to be more affordable. Previous studies on IgY-based antivenoms against snake and scorpion venoms have demonstrated that neutralizing antibodies can be raised by this method, albeit with lower neutralizing capacity than antibodies present in plasma-derived antivenoms [71]. Nevertheless, there has been a slightly increased interest in the use of egg-yolk-derived IgY antibodies for antivenom manufacture [72,73,74,75]. As an example, in a recent study, it was demonstrated using a rescue assay involving mice that the lethality of *A. australis hector* (*Aah*) venom could be neutralized with IgY antibodies. However, in addition to having the same drawbacks as equine IgGs in terms of polyclonality and batch-to-batch variation, avian-derived IgYs are phylogenetically even more distantly related to mammals [76] and still require the use of production animals for their manufacture. Therefore, it seems likely that the development of next-generation antivenoms based on recombinant monoclonal antibodies and antibody fragments may be a more promising research avenue for developing effective, safe, and cost-competitive antivenoms with defined therapeutic composition for future envenoming therapy.

#### 3.1.2. Recombinant Antivenoms

In the field of next-generation antivenoms, the development of phage display technology and recent advances in toxicovenomics have enabled researchers to pursue new therapeutic strategies with the purpose of creating better envenoming therapies [77]. These technology-driven approaches have made it possible to identify the most toxic, and thus medically relevant, components in a given venom, and to efficiently and systematically discover human monoclonal antibodies against these components. In turn, this has paved the way for developing recombinant antivenoms with anticipated improved safety profiles compared to conventional antivenoms. As an example, using phage display technology, the discovery of a human monoclonal single-chain variable fragment (scFv) antibody against *T. serrulatus* toxins, serrumab, was reported in 2012 [78]. This monoclonal antibody was demonstrated to have a high neutralizing capacity against β-toxins from *T. serrulatus*, while it was also capable of cross-neutralizing toxins from *C. suffusus suffusus* and *L. quinquestriatus*. More recently, in 2019, the field of recombinant scorpion antivenoms took another step forward when the discovery of a broadly-neutralizing monoclonal scFv antibody (scFv 10FG2) capable of neutralizing an estimated 13 different neurotoxins from different scorpion species belonging to the *Centruroides* genus was reported [79]. Using a rescue assay, this study demonstrated that scFv 10FG2 was capable of neutralizing 3 LD_50_s of freshly obtained whole venoms from five different scorpion species, belonging to the *Centruroides* genus, in 1:10 and 1:20 molar ratios of venom to antibody. The implementation of broadly-neutralizing antibodies in the formulation of recombinant antivenoms is a significant simplification in their pharmaceutical composition, since the inclusion of only a few broadly-neutralizing antibodies may be sufficient to neutralize the entire venoms of several different species. Reducing the product complexity of recombinant antivenoms is an important challenge to solve to enable cost-competitive manufacture of such medicines [80,81].

Next to scFvs, single-domain V_H_H antibodies, also known as nanobodies (Nb), are being intensively explored for their utility in relation to recombinant antivenoms. Nbs are native to the immune system of camelids and sharks and constitute the smallest natural antibody fragments known to date [82]. The low molecular mass of Nbs allows for their distribution into deep tissues throughout the body [83,84]. This property, in addition to their high ex vivo stability and low immunogenicity, makes them an interesting format for the development of next-generation scorpion antivenoms [5,85]. As an example of their utility, a preclinical study on a bispecific Nb, targeting AahI and AahII toxins from *A. australis* venom, demonstrated that this Nb was able to provide in vivo protection against 100 LD_50_s of intracerebroventricularly administered AahI (toxin to Nb molar ratio of 1:2) and 5 LD_50_s of subcutaneously administered whole venom. These results were significantly better than controls, in which equimolar amounts of traditional equine antibody fragments were unable to neutralize 2 LD_50_s of subcutaneously administered whole venom [86]. Finally, using a murine model, it was demonstrated that low molecular mass toxin–Nb complexes seemed to be quickly cleared through glomerular filtration, while uptake in the liver remained low [87]. Thus, these studies demonstrated that Nbs may possess promising pharmacokinetic and pharmacodynamic characteristics. Similar studies on *H. lepturus* and *Hottentotta saulcyi* with comparable results also support the notion that Nbs may find their utility for developing novel treatments for scorpion envenomings [83,88].

Finally, monoclonal IgGs are another recombinant antibody format that has been investigated for its ability to neutralize animal toxins. As an example, in vivo lethality studies assessing the neutralization capacity of several murine monoclonal IgGs have shown positive results for the neutralization of *A. australis* and *C. noxius* toxins [66]. In the future, however, it is likely that research in this area will focus more on human monoclonal IgGs, rather than non-human IgGs, as human IgGs have a range of benefits over heterologous IgGs. An overview of the benefits and drawbacks of different antibody formats can be found elsewhere [66].

## 4. Benefits of Scorpion Venom: Ongoing Research on Scorpion Toxins with Potential Therapeutic Applications

**Figure 4:**
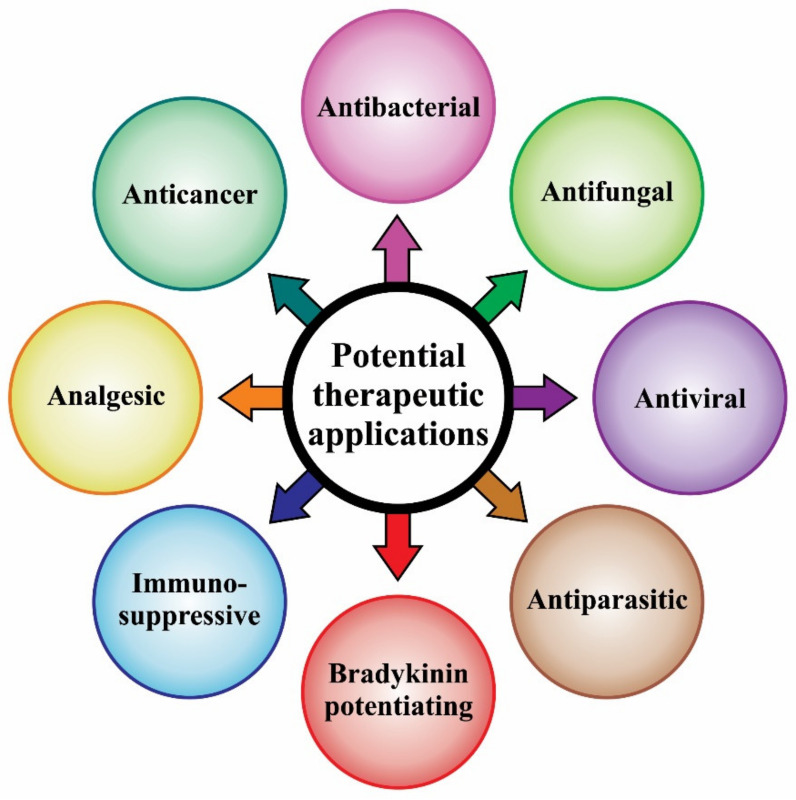
The potential therapeutic applications of scorpion venom compounds discussed in this article.

It is widely reported in the literature that scorpion venom is a rich source of bioactive compounds, and as such, their toxins are of interest to the pharmaceutical and biotech industries [89]. However, despite the fact that substantial research efforts are ongoing and the prospects for scorpion-derived therapeutic peptides are very promising, chlorotoxin is the only toxin from scorpion venom that has been taken into clinical trials [90]. Moreover, no scorpion toxin-based drug is currently found in the market [91]. In this section, potential applications of scorpion venom compounds, which have been the subject of therapeutic research, are presented (Figure 4), with a focus on results recently reported in the scientific literature. A comprehensive overview of such compounds, including older research reports, can be found elsewhere [12].

### 4.1. Antibacterial Effects

In the past century, antimicrobial drugs revolutionized the control of diseases caused by microorganisms, such as bacteria, fungi, viruses, and parasites. However, due to the global problem of antimicrobial resistance (AMR) development, new antimicrobial agents are crucially needed for the 21st century. These agents must be discovered at a rate that is sufficiently fast to combat the evolving rate of multidrug resistance (MDR) in microorganisms [92]. Natural product research holds promise for providing new molecules as a basis for novel antimicrobial drug development. In 1991, it was reported that the folding pattern of charybdotoxin, a KTx isolated from *L. quinquestriatus hebraeus* venom, was strikingly similar to that of the insect antibacterial component, defensin [93]. This discovery set the stage for studies on scorpion-derived antimicrobial peptides (AMPs), which have led to a large number of discoveries that may be of relevance for therapeutic applications. Comprehensive reviews on the different classes of AMPs found in the venom of several scorpion species can be found elsewhere [94,95]. Here, the focus will be directed only on more recent discoveries, with an overview of AMPs with bactericidal activity that have been reported in the last five years, which is summarized in Table 1.

**Table 1:** Scorpion-derived compounds with antibacterial activities. MDR: multidrug resistance.

Year	Scorpion Species	Antibacterial Agent	MW (S–S Bridge)	Target	Reference
2015	*A. aeneas*	AaeAP1	2016.18 Da (0)	*S. aureus*	[96]
		AaeAP2	1986.15 Da (0)		
2015	*C. margaritatus*	Cm38	2149 Da (2)	*Klebsiella pneumonia*	[97]
2015	*T. stigmurus*	Stigmurin	1795.22 Da (0)	Gram-positive bacteria including *S. aureus* and Methicillin-resistant *S. aureus* (MRSA)	[98]
2016	*M. gibbosus*	Low molecular mass chitosan *	3220 Da (0)	Bacterial species in general, including *Listeria monocytogenes*, *Bacillus subtilis*, *Salmonella enteritidis,* and the yeast *Candida albicans*	[99]
2016	*Scorpio maurus palmatus* (synthetic)	Smp-24	2578 Da (0)	Highest activity against Gram-positive bacteria, limited activity against *C. albicans*	[100]
		Smp-43	4654.3 Da (0)		
2017	*Isometrus maculatus*	Im-4	1714 Da (0)	Gram-positive bacteria	[101]
		Im-5	2803.7 Da (0)	Gram-positive and Gram-negative bacteria	
		Im-6	1707 Da (0)	Gram-positive bacteria	
2018	*T. obscurus*	ToAP2	9486 Da (0)	*Mycobacterium massiliense*	[102]
2018	*M. eupeus*	Meucin-49	5574.93 Da (?)	Gram-positive bacteria	[103]
		Meucin-18	2107.13 Da (0)	Gram-negative bacteria	
2018	*M. martensii*	Marcin-18	2135.63 Da (0?)	Gram-positive bacteria, including some clinical antibiotic-resistant strains	[104]
	*M. gibbosus*	Megicin-18	2068.04 Da (?)		
	*M. eupeus*	Meucin-18	2107.13 Da (0)		
2018	*Liocheles australasiae*	LaIT2	6628.2 Da (3)	Gram-negative bacteria	[105]
		N-LaIT2	3326 Da (?)		
2018	*T. stigmurus*	StigA6	1908 Da (0?)	Gram-positive and Gram-negative bacteria	[106]
		StigA16	1949 Da (0?)		
2019	*D. melici*	Red 1,4-benzoquinone: 3,5-dimethoxy-2-(methylthio)	168.15 Da	*S. aureus*	[53]
		cyclohexa-2,5-diene-1,4-dione *		*M. tuberculosis*, including an MDR strain	
		Blue 1,4-benzoquinone: 5-methoxy-2,3-bis(methylthio)cyclohexa-2,5-diene-1,4-dione *			
2017	*U. yaschenkoi*	UyCT1	1603.9 Da (0)	Gram-positive and Gram-negative bacteria	[107]
		UyCT3	1433.7 Da (0)		
		UyCT5	1442.7 Da (0)		
		Uy17	1369.43 Da (0)		
		Uy192	1459.98 Da (0)		
		Uy234	1986.19 Da (0)		
2019	*U. yaschenkoi*	Uy234	1986.19 Da (0)	MDR bacteria, including β-hemolytic *Streptococcus* strains	[108]
		Uy17	1369.43 Da (0)		
		Uy192	1459.98 Da (0)		
2017	*U. manicatus*	Um2	2034.56 Da (?)	Gram-positive and Gram-negative bacteria	[107]
		Um3	1577.23 Da (?)		
		Um4	1428.58 Da (?)		
		Um5	1508.82 Da (?)		
2019	*T. serrulatus* (hemolymph)	Serrulin	3564 Da (?)	Gram-positive and Gram-negative bacteria	[109]
2019	*A. australis hector*	G-TI	7390 Da (4, predicted)	*B. cereus*	[110]

The compounds mentioned in Table 1 are from the scorpion venom, unless otherwise specified. Whenever data on the number of disulfide bridges were not available for a compound, a question mark (?) is used. Non-peptidic compounds are marked with a star (*).

Recently, the spotlight has been put on the general and intrinsic multifunctionality of scorpion venom components, including AMPs [103]. For instance, native scorpion AMPs, UyCT3, and UyCT5 from *U. yaschenkoi* and an enhanced UyCT peptide (designated as D3) were demonstrated to be potential bioinsecticides and promising candidates for the engineering of aphid-resistant crops. When pea aphids (*Acyrthosiphon pisum*) that are known as severe agricultural pests were fed with these AMPs, the AMPs displayed activity against aphid bacterial symbionts and reduced the number of symbionts, leading to a reduction in pest survival and delay in pest reproduction [111]. Meucin-49 from *M. eupeus* also showed insecticidal activity in addition to having broad-spectrum activity against Gram-positive and Gram-negative bacteria [103]. The red and blue benzoquinones from *D. melici* are multifunctional components that, besides showing antibacterial activity, also exert cytotoxic effects on neoplastic cell lines. In mouse models of MDR tuberculosis infection, blue benzoquinone showed comparable activity to commercially available antibiotics, while it did not cause adverse side effects in healthy mice [53]. Similarly, the low molecular mass chitosan obtained from *M. gibbosus* had a strong inhibitory effect against the bacterium *L. monocytogenes* and the yeast *C. albicans*. In addition, its antibacterial activity against *B. subtilis* and *S. enteritidis* was higher than the antibiotic, gentamicin [99].

Despite the multifunctionality and desirable potent action against microbes, natural scorpion AMPs generally have cytotoxic effects on eukaryotic cells, which is an obstacle that must be overcome. To this end, protein engineering techniques have been used to improve the potency and spectra of antimicrobial activity of the natural scorpion AMPs [107,112,113]. Employing these techniques, it has been demonstrated that scorpion AMPs can be effectively used as scaffolds to design more specific and less harmful antibiotics [114,115]. In addition, combining low concentrations of fast-killing scorpion AMPs with classical antibiotics is another approach that can be pursued in order to circumvent their cytotoxic effects against eukaryotic cells [116]. All in all, using natural scorpion AMPs as scaffolds for the rational design of novel antimicrobial agents and mixed formulations of antibiotics opens a new window of research to be pursued in the future.

### 4.2. Antifungal Effects

The most important opportunistic fungal pathogens that are responsible for high mortality, especially in hospitalized and immunocompromised/critically ill patients, belong to the *Candida*, *Aspergillus*, *Cryptococcus*, and *Pneumocystis* genera [117]. It has been reported that the prevalence of invasive fungal infections has increased from 6.3% in 1999 to 20% in 2013 [118]. Among the aforementioned genera, *Candida* is the most common cause of fungal infections worldwide, and invasive candidiasis occurs in more than 100,000 patients every year [119]. Antifungal drug resistance among *Candida* species is increasingly reported, and the emergence of MDR *C. glabrata*, which can acquire resistance following exposure to antifungal agents, presents significant challenges in many medical centers [120]. Moreover, only three drug classes are licensed for monotherapy against *Candida* infections including azoles, polyenes, and echinocandins [120]. Therefore, new antifungal drug candidates from additional drug classes are sought after. A summary of the scorpion-derived antifungal agents reported in the last five years is found in Table 2.

Stigmurin, selected and synthesized based on a transcriptomic analysis of the *T. stigmurus* venom gland, exhibits both antibacterial and antifungal activity. It is effective against the Gram-positive bacterial species, *S. aureus*, including methicillin-resistant strains. Stigmurin has also been demonstrated to be effective against the fungi *C. albicans*, *C. krusei*, and *C. glabrata*, with low toxicity against healthy human erythrocytes [98]. These data suggested that stigmurin could be considered for the treatment of candidiasis. More recently, two analog peptides, StigA6 and StigA16, were designed from the original peptide that demonstrated improved antimicrobial and antifungal activity. These peptides could inhibit the growth of both Gram-positive and Gram-negative bacteria, as well as *C. albicans*, *C. krusei*, and *C. glabrata*, at lower minimal inhibitory doses compared to stigmurin [106]. StigA6 and StigA16 also showed high antiparasitic activity against *Trypanosoma cruzi* (see Section 4.4). This study demonstrated that rational design using scorpion toxins as scaffolds may be useful for obtaining leads with improved therapeutic features against a wide range of pathogens, including fungi.

### 4.3. Antiviral Effects

Few antiviral vaccines and drugs are commercially available against the more than 200 viruses known to infect humans [124], which is a situation that has been highlighted by the current SARS-CoV-2 pandemic and puts an emphasis on the importance of discovery and development of new antiviral agents. To this end, venomous animals are considered by many researchers as promising sources for such discoveries [124,125]. While some scorpion toxins show specific antiviral effects against just one type of virus, other toxins are active against several different viruses. Mucroporin-M1, a derivative of mucroporin from the *Lychas mucronatus* venom, presents antiviral activities against three RNA viruses (measles (MeV), severe acute respiratory syndrome-related coronavirus (SARS-CoV), and influenza H5N1). Binding assays demonstrated that there is a significant and specific interaction between immobilized mucroporin-M1 on CM5 biosensor chips and MeV. Following the mixing of 1 × 10^3^ plaque-forming units per milliliter (PFU/mL) of MeV with different concentrations of mucroporin-M1 and incubating the mixture for 1 h at 37 °C, two probable mechanisms of action were assessed. These assessments included measurements of the direct effects of mucroporin-M1 on the virus through the inhibition of MeV plaque formation and evaluation of the compound’s ability to compromise the infectivity of the virus through the suppression of MeV replication. The results showed that MeV infectivity could be inhibited almost completely by 10 µg/mL of mucroporin-M1 within 40 min. In a similar way, mucroporin-M1 showed inhibitory effects against both SARS-CoV and H5N1 pseudoviruses [126]. Later, it was demonstrated that mucroporin-M1 can inhibit the replication of the Hepatitis B virus through the activation of the mitogen-activated protein kinase (MAPK) pathway and downregulation of HNF4α in vitro and in vivo [127]. Given the dual inhibitory activities against viruses and bacteria, mucroporin-M1 may be considered as a lead compound for treating viral and bacterial co-infections. Mucroporin-M1 also serves as an example demonstrating the potential of peptides from scorpion venoms to be used as scaffolds for designing multifunctional antiviral agents.

Another example is the recombinant peptide, rEv37, from the scorpion *Euscorpiops validus*, which was been demonstrated to possess inhibitory effects against dengue virus type 2 (DENV-2), hepatitis C virus (HCV), Zika virus (ZIKV), and herpes simplex virus type 1 infections at non-cytotoxic concentrations. The inhibitory effects of rEv37 against DENV-2, HCV, and ZIKV infections were determined in the hepatoma cell line Huh7 via real-time fluorescent quantitative PCR for mRNA in the infected cells. rEv37 was able to reduce the level of DENV-2, HCV, and ZIKV infection at the mRNA level at a concentration of 10 µM by 91%, 97%, and 87%, respectively. Since the cellular entry processes of these four viruses are similar, it has been suggested that a specific molecular mechanism, in which the rEv37 peptide alkalizes acidic organelles to prevent low pH-dependent fusion of the viral membrane to the endosomal membrane, blocks the release of the viral genome from the endosome to the cytoplasm and thus restricts viral late entry [128]. The propensity to cause adverse reactions, lack of or low efficacy, and high price of the very few vaccines and therapeutics that are available against the aforementioned viruses [129,130] emphasize that rEv37 may be a relevant lead that possibly could be developed into an antiviral drug.

Smp76, a scorpine-like peptide from the venom of *S. maurus palmatus*, is another recent example of a scorpion-derived agent that is effective against different viruses. The recombinantly expressed peptide (rSmp76) can inhibit RNA replication and protein synthesis of DENV-2 and ZIKV in primary mouse macrophages, the human lung adenocarcinoma cell line (A549), the Huh7 cell line, and the human monocytic cell line (THP-1) in a dose-dependent manner. At a concentration of 10 mM, the inhibitory effects of rSmp76 were 75.7% against infections caused by DENV-2 (*TSV01*) and its more virulent strain (NGC), while for ZIKV infection, inhibition was evaluated to be 73.8%. Although the detailed molecular mechanisms of the rSmp76-induced inhibitory effects need to be elucidated, it seems that the mechanism of inhibition did not include direct inactivation of the viral particles. It has been suggested that rSmp76 suppresses an established viral infection by upregulating interferon-β expression through the phosphorylation of the interferon regulatory factor 3, which enhances type-I IFN responses and thus inhibits viral infection [131]. Since the achievement of viral clearance is very difficult, antiviral agents, such as rSmp76, that can suppress established viral infections are considered to be more efficient than traditional antiviral therapeutics, which exert their antiviral effects through the direct inactivation of viral particles or the inhibition of viral cell entry [131]. Enhancing the protective effects of host innate immunity, such as interferon (IFN) activation, by antiviral agents, such as rSmp76, may potentially circumvent the development of drug resistance and the effects of genetic variability in the viral genome [132]. These examples, selected from dozens of ongoing studies, demonstrate the potential of scorpion-derived peptides to be developed as antiviral therapeutics.

### 4.4. Antiparasitic Effects

Parasitic diseases are considered a health problem, particularly in developing countries, where people are frequently infected by parasites belonging to the genera, *Plasmodium*, *Trypanosoma*, and *Leishmania*, among others [133,134]. Since antiparasitic therapeutic agents available for clinical use are often toxic [135], there is an urgent need for the discovery and development of novel therapeutics [136].

Scorpion toxins have been demonstrated to possess inhibitory effects against a number of parasites. Scorpine, purified from *Pandinus imperator* venom, was the first isolated scorpion toxin that demonstrated antiprotozoan effects against *Plasmodium berghei* [137]. Later on, recombinantly expressed scorpine produced 98% mortality in the sexual stage of *P. berghei* and 100% reduction in *P. falciparum* parasitemia [138]. Similarly, meucin-24 and meucin-25, two linear NDBPs synthesized from a cDNA library of the *M. eupeus* venom gland, demonstrated antimalarial activity. Both peptides inhibited the development of *P. berghei* and killed intra-erythrocytic *P. falciparum* parasites at micromolar concentrations without harming mammalian cells [139], making them potential candidates for antimalarial therapies.

Scorpion-derived agents can be effective against other parasites as well. *Taenia solium* (pork tapeworm) is a parasite responsible for taeniasis (intestinal infection) and cysticercosis (tissue infection) in humans [140]. In 2010, *T. solium* cysticercosis was added to the list of major Neglected Tropical Diseases of the World Health Organization (WHO) [141]. *T. crassiceps* is another species of the Taeniidae family of tapeworms that, due to extensive antigen similarity with *T. solium*, functions as an experimental model to test and screen promising antigens before testing them in pigs [142]. It has been demonstrated using in vitro assays that Hge36, a naturally occurring truncated form of a scorpine-like peptide from the *Hoffmannihadrurus gertschi* venom, can reduce the viability of *T. crassiceps* larval cysts at submicromolar concentrations while having a minimal effect on human lymphocytes [143]. This study demonstrated that scorpion-derived agents may hold potential as therapeutic agents for human cysticercosis disease.

Human African trypanosomiasis (sleeping sickness) and American trypanosomiasis (Chagas disease) are induced upon infection with the protozoan parasites, *T. brucei* and *T. cruzi*, respectively. Being considered endemic in Latin America, Chagas disease is a potentially life-threatening illness that affects 6–7 million lives according to the WHO [144]. In an in vitro assay, it was recently demonstrated that stigmurin and its analogs, StigA6, StigA16, StigA25, and StigA31, show high antiparasitic activity against epimastigote forms of *T. cruzi* that is a form naturally found in the gut of infected insect vectors [106,113]. StigA6 and StigA16 have also been shown to have activity against trypomastigote forms of *T. cruzi*, which are mainly found in the blood of patients in the acute phase of Chagas disease. In addition, these peptides demonstrate higher antiparasitic activity at a lower concentration compared to benznidazole, which together with nifurtimox are currently available as Chagas disease medicines [106]. Therefore, StigA6 and StigA16 may be utilized in the development of therapeutics against Chagas disease.

It has been demonstrated that Leishmania parasites are sensitive to peptides with antimicrobial and ion channel inhibitory activity. Since scorpion venoms are rich sources of such peptides, Borges et al. demonstrated that *T. discrepans* crude venom and its main fractions (TdI, II, and III) could inhibit the growth of promastigote forms (the motile, long-elongated flagellated infective form of the Leishmania parasite that develops in the midgut of the sandfly) of *L. mexicana*, *L. braziliensis*, and *L. chagasi* that eventually led to parasite death in vitro [145]. Unfortunately, to the best of our knowledge, leishmanicidal activity of compounds from the venoms of other scorpion species/families has not been reported, and it should be investigated whether the leshmanicidal effects are restricted to the genus *Tityus*.

### 4.5. Bradykinin-Potentiating Effects

Bradykinin is a potent endothelium-dependent vasodilator peptide with hypotensive properties that belongs to the kinin group of proteins. The angiotensin-converting enzyme (ACE) inactivates bradykinin by degrading it [146,147]. The inhibition of ACE via bradykinin-potentiating peptides (BPPs), such as captopril, which is derived from a peptide found in the venom of the lancehead viper (*Bothrops jararaca*), has been established as a clinically approved strategy for preventing hypertension [148].

The multifunctionality of scorpion toxins is in the limelight once again regarding the bradykinin-potentiating effects of scorpion venoms. It has been demonstrated that the C-terminal fragment of BmKbpp, an AMP from *M. martensii* venom with antibacterial and antifungal activities, shows significant sequence similarity with the peptide K12, which is a known ACE inhibitor from *B. occitanus* venom. In an in vitro assay using guinea pig ileum segments of nearly 3 cm in length, both BmKbpp whole peptide and its C-terminal fragment (BmKbpp-C) demonstrated bradykinin-potentiating activity at a concentration of 50 nM. However, BmKbpp whole peptide and BmKbpp-C were less potent than peptide K12, with BmKbpp-C being more active than the whole peptide [149]. The sequence similarity between a fragment of a toxin and BPPs is also observed for *T. serrulatus* venom peptides. The N-terminal of Ts3, an α-toxin acting on voltage-gated sodium channels, demonstrated a striking sequence similarity with Ts10 (former Peptide T) that is a known BPP [150]. Ts10 was originally reported as an ACE inhibitor, since it could inhibit the ACE-catalyzed hydrolysis of bradykinin [151]. Later, using male Wistar rats and their aortic rings, in vitro and in vivo assays demonstrated that the N-terminal of Ts3 (Ts3_1-14[C12S]_) and Ts10 were not able to directly inhibit ACE activity; instead, they induced a strong vasodilatory effect that could be reversed in the presence of the nitric oxide (NO) synthase inhibitor, N(ω)-nitro-L-arginine methyl ester (L-NAME). This suggests that Ts10 and Ts3_1-14[C12S]_ play their role by activating molecular targets in the vascular endothelium, which leads to NO production and eventually vasodilation [150]. In addition, it has been reported that *T. serrulatus* hypotensins (TsHpt-I, II, III, and IV) and TistH from *T. stigmurus* also potentiate bradykinin through improvement of the endothelial function and NO release in rats [152,153]. These cases show that scorpion peptides can potentiate bradykinin through mechanisms other than ACE inhibition.

Besides vasodilation and hypotension, scorpion-derived BPPs play important roles in other physiological processes. It is estimated that a considerable number of cancer patients receive radiation therapy during their course of illness [154]. However, radiation therapy might lead to side effects, including radiation-induced heart disease (RIHD) in patients having lymphoma, breast, lung, and esophageal cancer [155,156]. It has been demonstrated that BPPs obtained from *L. quinquestriatus* improved cardiomyopathy induced by γ-radiation in rats, probably by acting as a scavenger of free radicals to protect the heart from negative effects derived from radiation exposure [157].

### 4.6. Immunosuppressive Effects

Several scorpion toxins can modulate the immune system [158]. Indeed, the contribution of released inflammatory mediators (e.g., cytokines, eicosanoids, and reactive oxygen species) and activation of the complement system is well explored in the envenoming pathophysiology following scorpion stings [57,158,159]. For instance, an increase in the regulatory cytokines, interleukin (IL)-10 and IL-4, has been observed in experimental envenomings by *A. australis hector* and *C. noxius*, as well as in real human envenomings by *T. serrulatus* [160,161,162,163]. Although most of the studied scorpion toxins exhibit pro-inflammatory effects and activate the immune system [20,58,160,164,165], a few of them demonstrate potential therapeutic applications by controlling the immune responses and acting as immunosuppressive agents.

The most studied class of immunosuppressive scorpion toxins is the blockers of voltage-gated potassium channel type 1.3 (K_v_1.3). Although many cells express K_v_1.3, most of the studies have focused on effector memory T cells (T_EM_) due to the high expression profiles of K_v_1.3. The T_EM_ cells are a subpopulation of T cells regarded as an attractive pharmacological target because of their role in the development of autoimmune diseases [166]. A recent review covering the structure and function of these channels, as well as the therapeutic implications of blocking K_v_1.3 using toxins derived from scorpion venom, has summarized the studies of more than 60 scorpion toxins from the *Androctonus*, *Buthus*, *Mesobuthus*, *Lychas*, *Parabuthus*, *Leiurus*, *Centruroides*, and *Tityus* genera [167].

Beside K_v_1.3 channels, other ion channels, such as K_v_3.1 and K_v_2.1, have also been demonstrated to be important for T-cell activation and function [168,169]. Pucca et al. described Ts6 and Ts15, from *T. serrulatus*, which block K_v_2.1 and inhibit the proliferation and function of different T-cell subpopulations in vitro. The study also showed that Ts15 was capable of inhibiting delayed-type hypersensitivity (DTH) response in vivo, indicating the potential of the peptide to be developed into a treatment for autoimmune diseases [169]. Another study performed by Xiao et al. described the immunosuppressive and anti-inflammatory properties of St20, a disulfide-bridged α-KTx found in the venom of *Scorpiops tibetanus*. In vitro functional studies showed that this peptide was able to inhibit the expression of the cell surface marker CD69, as well as the secretion of IL-2, tumor necrosis factor (TNF)-α, and IFN-γ in activated human T cells. In vivo experiments using a rat autoimmune disease model showed that DTH was ameliorated in the presence of St20 [170]. Thus, new immunosuppressive therapeutic drugs may be derived from scorpion venom toxins, which can be optimized in regard to structure and function, possibly facilitating the future use of such agents in clinical settings.

### 4.7. Analgesic Effects

Generally, scorpion stings are reported as very painful events. Most known scorpion toxins are known to modulate voltage-gated ion channels (mainly sodium and potassium channels) [20]. Voltage-gated sodium (Na_v_) channels play a key role in nociception (pain) [171]. The Na_v_ channels comprise a family of nine homologous α-subunits (Na_v_1.1–Na_v_1.9), which together with β-units (β1–β4) generate the ion-conducting pore [172]. However, only four Na_v_ channel subtypes are involved in pain: Na_v_1.1, Na_v_1.6, Na_v_1.7, and Na_v_1.9 [173,174,175,176]. Throughout the last decades, scorpion toxins capable of inducing pain mediated by these channels have been widely explored [20,177,178,179,180]. Most recently, two peptides, Hj1a and Hj2a, have been isolated from the *Hottentotta jayakari* venom that are potent agonists of Na_v_1.1. Demonstrating dual α/β activity by modifying both the activation and inactivation properties of the channel, Hj1a and Hj2a may be used as alternative tools for developing selective Na_v_1.1 modulators for the treatment of epileptic diseases, such as Dravet syndrome [181]. In addition, scorpion toxins that induce pain, mediated by different ion channels, such as voltage-gated potassium channel 4.2 (K_v_4.2) [182] and TRPV1 [35,183], have also been encountered.

Moreover, scorpion toxins capable of controlling pain (i.e., analgesics) have been reported in the literature. Many of these analgesic toxins are not toxic to mammals, as they belong to a group of insect-specific neurotoxic α or β-toxins that interact with Na_v_, K_v_, and/or Ca_v_ pathways [114,184,185,186,187,188]. During the last two decades, over 20 scorpion venom-derived peptides and proteins have been reported to exert anti-nociceptive effects in vitro and in vivo. Due to the absence of toxicity in mammals and comparable effects to standard of care medications, such as carbamazepine, most of the scorpion-derived proteins are intriguing agents that could be used for future development of analgesics. The scorpion *M. martensii* (previously known as *B. martensii* Karsch) has been thoroughly studied as the source of more than 15 analgesic peptides [189,190]. Analgesic properties have also been reported in *A. mauretanicus mauretanicus* (AmmVIII, α-anatoxin), *L. quinquestriatus quinquestriatus* (LqqIT2, β-toxin), *H. laoticus* (Hetlaxin, α-toxin), *B. occitanus tunetanus* (BotAF, β-toxin), and *T. serrulatus* (TsNTxP) (Table 3). However, there is still much unexplored venom territory for future discovery in the field of analgesic venom components.

In 2019, Rigo et al. reported the presence of anti-nociceptive effects of a non-toxic protein from *T. serrulatus*, TsNTxP [191]. This protein is described to be structurally similar to Na_v_-modulating neurotoxins, such as Ts7. However, TsNTxP is non-toxic to animals. Effects of TsNTxP were studied in 184 adult male and female Swiss mice in regard to acute and neuropathic pain. The results demonstrated that TsNTxP has potent anti-nociceptive properties in both models, which is potentially due to a substantial reduction of glutamate release. These results, combined with the lack of acute adverse effects, suggest that TsNTxP may possibly be utilized in future pain treatment.

**Table 3:** Summary of known anti-nociceptive scorpion toxins.

Toxin Name	Scorpion Species	MW (S–S Bridge)	Target	Reference
BmK AS	*M. martensii*	7701 Da (4)	TTX-R (Na_v_1.8, 1.9), TTX-S (Na_v_1.3); reduction of neural excitability; skeletal muscle RyR	[192,193,194,195]
BmK IT2	*M. martensii*	6650 Da (4)	TTX-R and TTX-S Na_v_	[192,196]
BmK IT-AP	*M. martensii*	8157 Da (4)	N/A	[197]
BmK dITAP3	*M. martensii*	6740 Da (4)	N/A	[187]
BmK AEP/BmK ANEP	*M. martensii*	6738 Da (4)	Na_v_1.1, Na_v_1.3, Na_v_1.6, Na_v_1.7	[198,199,200]
BmK AS1	*M. martensii*	7712 Da (4)	TTX-R and TTX-S Na_v_, skeletal-muscle RyR-1	[201]
BmK AGAP	*M. martensii*	7281 Da (4)	Prevention of peripheral and spinal MAPKs expression; Decrease of spinal c-Fos expression; Na_v_1.7, Na_v_1.8, Na_v_1.4, Na_v_1.5; Ca_v_	[185,202,203,204,205]
BmK Ang P1	*M. martensii*	8141 Da (4)	N/A	[206]
BmK Ang M1	*M. martensii*	7040 Da (4)	Na_v_, K_v_	[114,207]
BmK(M)9	*M. martensii*	7106 Da (4)	Na_v_1.4, Na_v_1.5, Na_v_1.7	[208,209]
BmK AGP-SYPU1	*M. martensii*	7227 Da (4)	N/A	[210]
BmK AGP-SYPU2	*M. martensii*	7457 Da (4)	Na_v_	[211,212]
BmK AGAP-SYPU2	*M. martensii*	7253 Da (4)	Na_v_ (suspected)	[213]
BmK-YA	*M. martensii*	871 Da (0)	µ, κ, δ-opioid receptor	[188]
BmKBTx	*M. martensii*	6800 Da (3)	Na_v_1.7	[214]
BmNaL-3SS2	*M. martensii*	7338.26 Da (3)	Na_v_1.7	[214]
AmmVIII	*A. mauretanicus mauretanicus*	7383 Da (4)	Na_v_1.2, endogenous opioid system, no data on other Na_v_s yet	[186]
LqqIT2	*L. quinquestriatus quinquestriatus*	6845 Da (4)	Endogenous opioid system, no data on other Na_v_s yet	[186]
TsNTxP	*T. serrulatus*	6702 (4)	N/A (possibly glutamate release)	[191]
BotAF	*B. occitanus tunetanus*	7446 Da (4)	Peripheral or spinal mechanisms	[198]
Hetlaxin	*H. laoticus*	3665 Da (4)	K_v_1.3, K_v_1.1	[184,215]

### 4.8. Anticancer Effects

The discovery of specific and selective anticancer drugs that can directly act on tumors, display a synergistic effect with existing chemotherapeutics, or function as cargoes for drugs with low bioavailability is significantly on the rise [216]. Chlorotoxin (CTx) from *L. quinquestriatus* venom is a molecule that interacts with chloride channels. CTx was the first scorpion-derived agent that demonstrated inhibitory effects on glioma cell migration and invasion. It also exhibited the advantage of being able to penetrate deep into tumor tissue [217,218]. Since the discovery of CTx, the list of scorpion crude venoms and isolated toxins with anticancer activity has been growing rapidly, hence a comprehensive review of all reported compounds exceeds the scope of this review, but can be found elsewhere [216,217,218,219]. Here, we present a few cherry-picked recent studies with the most significant findings.

*T. serrulatus* crude venom was tested in 2019 for possible anticancer effects against the SiHa and HeLa cervical cancer cell lines, and the venom was shown to induce apoptosis in HeLa cells [220]. Wang et al. had previously obtained similar results with the crude venoms of *H. liangi* and *M. martensii*. The two venoms were tested for potential anticancer effects toward HeLa cells, and both venoms showed dose-dependent anti-proliferative and apoptosis-inducing effects through upregulation of the CDK-inhibitor, p21. However, neither of the venoms showed significant effects on non-cancer HUVEC-21 cells, suggesting specificity toward cancer cells [221]. Additionally, the venoms of *A. crassicauda* and *L. quinquestriatus* have been examined using breast (MDA-MB-231) and colorectal (HCT-8) cancer cell lines [222]. This examination revealed that the venoms exhibited significant time and dose-dependent cytotoxicity, and that they caused an increase in the number of apoptotic cells and reactive oxygen species for both cancer cell lines when the cell lines were subjected to the venoms. The observed arrests in the cell cycle could be an indication of tumor suppressor p21 upregulation and could, hence, suggest selectivity toward cancer cells. Anticancer properties have been recently associated to the crude venom of *Rhopalurus junceus* and a mix of five peptides from the same venom [223,224]. Despite generating promising results, further investigations on the aforementioned scorpion crude venoms are needed to characterize the effective anticancer constituents among other venom components.

In 2018, Li et al. constructed a scorpion venom library of *A. australis* and *A. mauretanicus* that led to the discovery of a highly potent novel anticancer peptide from *A. mauretanicus* named Gonearrestide. This peptide was subsequently tested against several colorectal cancer cell lines (DLD-1, Hke3, Dks8, and HCT116) and the glioma cell line, U-251. Extensive in vitro, in vivo, and ex vivo studies on HCT116 cells demonstrated that this peptide possessed high specificity toward cancer cells, could significantly arrest the cell cycle in the G1 phase, and could thus strongly inhibit tumor growth. Additionally, proliferation and cytotoxicity studies with the non-cancer human epithelial cell lines FHC (colon), MCF-10A (breast), and human erythrocytes only identified negligible off-target effects of Gonearrestide [225]. Another study by BenAissa et al. investigated the activity of the negatively charged fractions of *A. australis* venom against DU145 prostate cancer cells and successfully identified strong anti-proliferative effects mediated by the Na_v_1.6-directed peptide AaHIV [226]. However, regardless of its potent anti-proliferative effects, this peptide was not able to inhibit cell migration. Another study on AGAP and AGAP-SYPU2 from *M. martensii* venom (see Section 4.7 for their analgesic effects) demonstrated that these two peptides possessed in vivo antitumor properties in mouse Ehrlich ascites tumor models and mouse S-180 fibrosarcoma models [213,227]. Furthermore, AGAP showed strong anticancer effects, including the inhibition of stemness, epithelial–mesenchymal transition, migration, and invasion toward MCF-7 and MDA-MB-231 breast cancer cells [228]. It also inhibited the voltage-gated proton channel Hv1 [229], which has been investigated as a possible target for cancer therapy and has been extensively reviewed elsewhere [230]. Two studies on a third *M. martensii* peptide, BmKn2, indicated that this peptide could selectively induce apoptosis in cancerous human oral cells, while normal cells were affected to a much lesser extent [231,232]. Another interesting study highlights a newly discovered short (14 residues) peptide from *B. occitanus tunetanus*, RK1, with potent anticancer effects toward glioblastoma (U87) and melanoma (IGR39) cancers [233]. While showing no apparent in vivo neurotoxicity toward intracerebroventricularly injected mice, RK1 was demonstrated to possess cytotoxicity against U87 and IGR139 cells in vitro, and it was able to inhibit cell proliferation and migration of these two cancer cell lines. RK1 also inhibited angiogenesis in a chicken chorioallantoic membrane model. A cell-penetrating peptide (CPP) from *T. serrulatus* venom, named CPP-Ts, is the final promising member of the long list of scorpion-derived agents with anticancer effects discussed here. Using a CPP-Ts-derived peptide (subpeptide^14–39^), a study demonstrated that this peptide has selective internalization properties in specific cancer cell lines, such as SK-MEL-188, HEP G2, Caco-2, MDA-MB-231, A549, and DU 145, which make it a potential intranuclear delivery tool for cancer cell targeting [34]. These handful of studies from the growing body of recently published papers indicate the potential of utilizing scorpion venoms as a source for discovering new cancer therapeutics.

## 5. Conclusions

The large diversity of scorpion venom components has fueled a wide range of studies on these molecules, from toxicology to antivenom development and therapeutic applications. In particular, therapeutic applications of scorpion venom compounds have attracted a lot of attention due to the urgent need for either finding or improving treatments against a broad spectrum of diseases. The emergence and spread of superbugs (AMR microorganisms) represent an increasingly serious threat to global public health, which is projected to get much worse in the years ahead. Therefore, the exploration of the utility of novel bioactive molecules, scaffolds, and mechanisms of action represents a potentially powerful approach to develop new antimicrobial therapeutics and diagnostic tools for current and future diseases. Dozens of scorpion-derived bioactive molecules have been shown to possess promising pharmacological properties, of which around 100 have been mentioned in this review. These pharmacological properties of scorpion-derived bioactive molecules include antimicrobial, immunosuppressive, bradykinin-potentiating, analgesic, and anticancer effects among others. In addition to chlorotoxin, which has already entered clinical trials, the CPP-Ts peptide (which is a potential intranuclear delivery tool for targeting cancer cells) is likely to be a molecule receiving significant scientific interest in the future. However, before venom-derived biotherapeutics can be introduced to the market, a number of technological issues must be overcome, including obtaining access to material (venoms and toxins), characterizing isolated venom components, establishing manufacturing approaches for novel compounds, and reducing the potential propensity to cause adverse effects, especially for long-term therapy. Nevertheless, the scientific literature reviewed here shows several examples of promising scorpion venom-derived proteins and peptides that may be used as leads for the development of new biotherapeutics. Thus, if the data observed in vitro and in preclinical models translates well to the clinical setting, there may indeed be great promise in exploiting the benefits of scorpion toxins.

## Figures and Tables

**Table 2:** Reported work on scorpion derived antifungal agents.

Year	Scorpion Species	Antifungal Agent	MW (S–S Bridge)	Target	Reference
2015	*T. stigmurus*	Stigmurin	1795.22 Da (0)	*C. albicans*, *C. krusei*, and *C. glabrata*	[98]
2015	*A. aeneas*	AaeAP1	2016.18 Da (0)	*C. albicans*	[96]
		AaeAP2	1986.15 Da (0)		
2016	*T. stigmurus*	Hypotensin TistH	2700 Da (0)	*C. albicans*, *C. tropicalis* and *Aspergillus flavus*	[121]
2016	*T. obscurus*	ToAcP, ToAP1, ToAP2, ToAP3, ToAP4	? (0)	*Cryptococcus neoforman* and *Candida species*	[122]
2017	*T. serrulatus*	Ts1	8300 Da (3)	*A. nidulans*	[123]
2018	*T. stigmurus*	StigA6	1908 Da (0?)	*C. albicans*, *C. krusei*, and *C. glabrata*	[106]
		StigA16	1949 Da (0?)		
2019	*T. serrulatus* (hemolymph)	Serrulin	3564 Da (0)	*A. niger* and *C. albicans*	[109]

The compounds mentioned in Table 1 are from the scorpion venom, unless otherwise specified. Whenever data on the molecular weight and/or the number of disulfide bridges were not available for a compound, a question mark (?) is used.
